# An Optimized Trichloroacetic Acid/Acetone Precipitation Method for Two-Dimensional Gel Electrophoresis Analysis of Qinchuan Cattle Longissimus Dorsi Muscle Containing High Proportion of Marbling

**DOI:** 10.1371/journal.pone.0124723

**Published:** 2015-04-20

**Authors:** Ruijie Hao, Camus Adoligbe, Bijie Jiang, Xianlin Zhao, Linsheng Gui, Kaixing Qu, Sen Wu, Linsen Zan

**Affiliations:** 1 College of Animal Science and Technology, Northwest A&F University, Yangling, Shaanxi, P.R. China, 712100; 2 National Beef Cattle Improvement Center, Northwest A&F University, Yangling, Shaanxi, P.R. China, 712100; Università della Calabria, ITALY

## Abstract

Longissimus dorsi muscle (LD) proteomics provides a novel opportunity to reveal the molecular mechanism behind intramuscular fat deposition. Unfortunately, the vast amounts of lipids and nucleic acids in this tissue hampered LD proteomics analysis. Trichloroacetic acid (TCA)/acetone precipitation is a widely used method to remove contaminants from protein samples. However, the high speed centrifugation employed in this method produces hard precipitates, which restrict contaminant elimination and protein re-dissolution. To address the problem, the centrifugation precipitates were first grinded with a glass tissue grinder and then washed with 90% acetone (TCA/acetone-G-W) in the present study. According to our result, the treatment for solid precipitate facilitated non-protein contaminant removal and protein re-dissolution, ultimately improving two-dimensional gel electrophoresis (2-DE) analysis. Additionally, we also evaluated the effect of sample drying on 2-DE profile as well as protein yield. It was found that 30 min air-drying did not result in significant protein loss, but reduced horizontal streaking and smearing on 2-DE gel compared to 10 min. In summary, we developed an optimized TCA/acetone precipitation method for protein extraction of LD, in which the modifications improved the effectiveness of TCA/acetone method.

## Introduction

Proteomics, presented in 1994 [1], creates new possibilities in the elucidation of biopathomechanisms and the discovery of novel biomolecular markers [2]. Up to now, the emergence of gel-free approaches, including protein antibody arrays [3] and multi-dimensional liquid chromatography with stable isotopic labeling [4], has further enriched our research tools in proteomic analysis. Nonetheless, the two-dimensional gel electrophoresis (2-DE), developed in the mid 70s [5,6], is still one of the most widely used separation techniques for its affordable price, robustness, resolution, and ability to separate entire and intact proteins [7,8]. The initial sample extraction is essential for 2-DE separation of proteins and subsequently affects the following analysis. The ideal sample preparation procedure should reproducibly capture the most comprehensive repertoire of proteins without any artifactual modification, proteins loss, degradation or non-proteinaceous contamination [2,9–11]. However, the intrinsic difficulty lies in the fact that the vast diversity of protein properties makes it unfeasible to use a single sample preparation protocol that sufficiently applies to any given biological system [2].

Longissimus dorsi muscle (LD) in beef cattle contains very high proportion of marbling [12] that is important for taste and tenderness of beef. The more insight into the distinct protein expression pattern of LD is very significant to reveal the molecular mechanism behind intramuscular fat accumulation. However, the 2-DE analysis of these tissues is very difficult due to the high concentration of lipids and nucleic acids [13] that can cause such problems as smearing, horizontal and vertical streaking in 2-DE gel images [13–16]. TCA/acetone precipitation method is commonly used to remove interfering compounds. Whereas, the solid precipitates caused by high speed centrifugation are difficult to crush, which restrict contaminant elimination and protein re-dissolution, leading to poor 2-DE gel [17,18]. To date, the improved methods of sample preparation for recalcitrant tissues are more available [14,19–26], however, there are very limited published papers focusing on protein extraction of LD tissue in beef cattle [12], in spite of increasing importance of cattle LD proteomics [27–29].

The present work was carried out to develop an effective protein extraction method for LD, which should maximumly eliminate interfering substances and avoid protein loss caused by TCA/acetone precipitation. In doing this, we incorporated into the traditional TCA/acetone precipitation two novel steps including precipitate grinding using a glass tissue grinder and adopting 90% acetone as washing solution. The effect of two additional steps on protein yield, image quality and spot patterns was evaluated by comparison with the following methods: i) direct protein extraction by lysis buffer; ii) classical TCA–acetone [30–32]; iii) another commonly used TCA–acetone [24,33,34], where the sample was first extracted in an aqueous buffer before being precipitated (TCA/acetone-B). In addition, we also focused on the protein drying after acetone washing, and made a detailed comparison of 2-DE results between 10min and 30min air-drying.

## Material and Methods

### Ethics statement

This study was performed with the approval of the Experimental Animal Manage Committee (EAMC) of Northwest A&F University. Animals were treated as the guidelines of EAMC.

### Animals and muscle samples

Chinese Qinchuan cattle were fattened in the National Beef Cattle Improvement Center in China, and slaughtered after stunned at 27 months of age. LDs between 12 and 13 ribs were dissected and collected within 20 min after slaughter and snap-frozen in liquid nitrogen pending protein extraction. For each protocol assayed, three samples corresponding to three randomly selected sets of individuals were prepared.

### Protein extraction

Protein extraction was based on the protocols described by Bouley et al. [35], Gorg et al. [31], Liu et al. [34]and Islam et al. [23]with a few modifications. Frozen LD samples (~50 mg) were crushed in a pre-cooled mortar with liquid nitrogen until fine powder was formed. The LD powder was then placed in a 1.5 ml pre-chilled eppendorf tube and subjected to the following extraction procedures: (i) Direct extraction by lysis buffer: proteins were extracted by adding 500μL of extraction buffer containing 7M urea, 2M thiourea, 65Mm DTT, 2% CHAPS, 2% sb3–10, 0.5% Bio-Lyte pH 3–10, 20mM tris, and 1% protease inhibitor cocktail solution (BS386, Sangon, China) [35]. The sample was sonicated on ice using a probe sonicator (VCX 150; Sonics & Materials Inc. USA) in 8×10s bursts with 10s intervals, and then vortexed every 15min for 30s, 4 times in total before centrifugation. (ii) Classical TCA–acetone extraction: freshly prepared 10% TCA in 0.2% DTT-containing acetone (1.3 mL) was added to 50 mg of sample. The solution was then placed at -20°C overnight, followed by centrifugation in a pre-cooled rotor at 3,5000g for 30 min at 4°C using a Beckman allegratm 64R preparative centrifuge (Palo Alto, CA, USA). After discarding the supernatant, the pellet was crushed using a clean pre-chilled grinding rod and resuspended in 1.3 mL of 0.2% DTT in acetone, then placed at -20°C for 1 h followed by 40,000g centrifugation, with the precipitates recovered and rinsed two more times [31]. Residual acetone in samples was removed by air-drying for 30 min at 20°C. Protein precipitates were then solubilized as done previously in step i. (iii) Another TCA/acetone extraction (TCA/acetone-B): 50 mg of sample dealt with as step i, homogenates were then centrifuged at 40,000g at 4°C for 1 h, and the supernatants (below the fat cake) were collected and precipitated by 10% trichloroacetic acid, with the next procedures following the principle of step ii [23,34]. (iv) Modified TCA/acetone extraction (TCA/acetone-G-W): the procedures were the same as step iii, except for the following: the solid centrifugation pellet was transferred into a pre-chilled glass tissue grinder (Sangon, China) and briefly grinded into emulsion via pre-chilled acetone before washing, after which the protein powder was washed with 90% acetone containing 0.2% DTT (–20°C) in place of 100% acetone. (v) The sample was treated according to step iv with a change of air-drying period (10 min instead of 30 min).

After the procedures mentioned above, the samples were centrifuged at 40,000g for 1 h and the protein concentration was quantified with the method of Bradford [36], using bovine albumin as standard. For every protocol above, three replicates were performed, each consisting of an independent protein extraction from different LD samples (biological replicates).

### 2-D electrophoresis, gel staining, image capture and analysis

Precast 17 cm pH 3–10 nonlinear (Bio-Rad) strips were rehydrated for 16 h with 300 μl buffer (200 μg protein) of 7M urea, 2M thiourea, 2% CHAPS, 2% sb3–10, 65mM DTT, 0.2% ampholytes (Bio-Lyte 3/10, Bio-Rad), and 0.005% bromophenol blue. Electrofocusing (Bio-Rad Protean IEF Cell system) was carried out at 20°C with a gradually increasing voltage: 50 V for 90 min, 100 V for 30 min, 250 V for 30 min, 500 V for 30 min, 750 V for 30 min, 1000 V for 30 min, 1000–10,000 V for 5 h, and to 60,000 VH with a maximum voltage of 10,000 V. After IEF, the gel strips were rinsed with SDS-PAGE electrophoresis buffer to remove residual rehydration solution and mineral oil, then reduced for 15 min in 4 mL equilibration buffer containing 0.375M Tris-HCl (pH 8.8), 6M urea, 20% v/v glycerol, 2% w/v SDS, and 2% w/v DTT, and alkylated in the above buffer modified by including 2.5% w/v iodoacetamide instead of DTT [37]. The strips were finally transferred onto 12% SDS-polyacrylamide gels sealed with 0.5% low metling agarose (Bio-Rad PROTEAN Plus Dodeca Cell). Electrophoresis ran at 5 mA/gel for the first 2 h [38], followed by 20 mA/gel until the dye front reached the bottom of the gel (about 12.5 h). Gels were silver stained as previously described [39,40] and then scanned by UMAX Powerlook 2100XL-USB ImageScanner (TAIWAN). Image and data analysis of the gels were performed with PDQuest software (Version 8.0; Bio-Rad).

### Statistics

Statistical analysis was performed using the SPSS version 16.0. Comparisons of mean values (protein yield, the number of protein spots) from different extraction procedures were made by ANOVA test. Multiple discriminant analysis (MDA) was conducted to test whether the 2-DE gels from different extraction methods were method-specific.

## Results and Discussion

One-step extraction by lysis buffer [35,41–44] is considered as the simplest and the most straightforward procedure, nevertheless it is not the best choice for complex tissue. In order to justify contaminant elimination from LD, we evaluated the effect of one-step direct extraction method as well as two traditional TCA/acetone methods on 2-DE, which served as controls for our modified method.

Muscle extracts were compared for protein concentration using the Bradford assay [36]. As indicated in Table 1, No significant difference in precipitated protein mass was found between methods (30 min air-drying). Protein concentration of direct extraction method was the highest of all, but with no significant difference to TCA/acetone-G-W (Table 1). These results implied that the pellet loss from additional hand grinding was puniness in TCA/acetone-G-W. The ratio of protein concentration to dried precipitate in TCA/acetone-G-W was significantly higher than other methods, suggesting less dried precipitate yielded higher protein concentration. In other words, the additional grinding improved solubility of proteins in agreement with what has been previously reported [23,45,46].

**Table 1:** Protein yields associated to the different protein extraction protocols.

Protein yield	Direct extraction by Lysis buffer(30min air-drying)	TCA/acetone (30min air-drying)	TCA/acetone-G-W (30min air-drying)	TCA/acetone-B (30min air-drying)	10min air-drying (TCA/acetone-G-W)
Dried precipitate (mg/50mg tissue ± SD)	\	15.67±1.53^B^	13.67±1.53^B^	14.00±2.00^B^	29.33±3.06^A^
protein concentration (mg/ml± SD)	11.23±0.61^a^	7.70±0.92^b^	9.57±1.11^ab^	7.63±1.06^b^	10.40±1.93^ab^
protein concentration /Dried precipitate (mean± SD)	\	0.49±0.05^bBC^	0.70±0.06^aA^	0.55±0.06^bAB^	0.35±0.04^cC^

Values followed by different letters are significantly different in a row (capital: p<0.01, lowercase: p<0.05).

**Fig 1:**
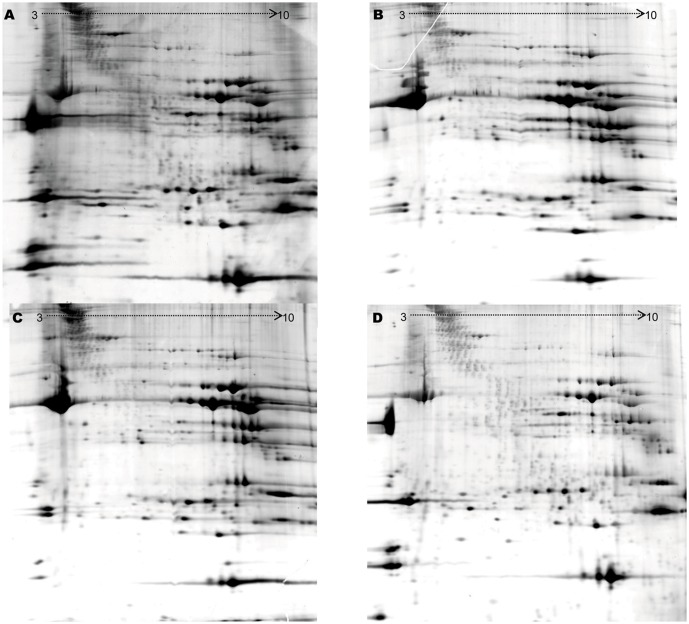
Representative 2-DE maps of LD protein extracted using different methods. A: one-step extraction by lysis buffer; B: TCA/acetone; C: TCA/acetone-B; D: TCA/acetone-G-W. The modified procedure of TCA/acetone-G-W gave the best 2-DE gel with a clear background and minimal streaking and smearing.

2-DE gels produced by direct extraction, TCA/acetone, TCA/acetone-B and TCA/acetone-G-W were presented in Fig 1. Highest resolution, least horizontal streaking, best spot focusing and most well resolved spots (1068±32, C V = 0.03) were observed in TCA/acetone-G-W, which suggested that contaminants had been effectively removed from LD protein extracts. To our knowledge, direct extraction of protein by lysis buffer is viewed as the simplest procedure, which can avoid the protein loss caused by precipitation [21,43]. However, in our result this method yielded smaller amounts of protein, and showed obvious horizontal streaking and smearing in the acidic range. This method protected the integrality of proteins to the maximum, however, more lipids and nucleic acids which interfered with protein focusing and separation also remained [12,47]. Furthermore, proteases, which were hard to be neutralized by protease inhibitor cocktails alone [48], hydrolyzed some proteins. It has been confirmed that direct extraction method was not applicable for LD due to high fat and nucleic acid contents. Notably, application of TCA/acetone improved gel resolution with smearing and streaking reduced. However, the number of protein spots (559±35, C V = 0.06) was still not satisfying. Presumably it may be ascribed to protein loss [20], because 40,000g centrifugation generated insoluble substances as a sediment in the tube after the step of protein re-dissolution. The survival of basic streaking suggested that vortexing solely was not sufficient to disperse precipitate for complete contaminant removal. TCA/acetone-B further released spot trailing, and visualized more distinct spots (631±34, C V = 0.05). Unfortunately, the horizontal streaking in the basic region still existed. A similar result has been obtained by Nazrul Islam et al revealing that TCA/acetone-B method could cause part of contaminants co-precipitate with proteins, disturbing protein separation in 2-DE [23]. Furthermore, Saravanan et al have got worse 2-DE gel using TCA/acetone-B than TCA/acetone in tomato proteomics research [24]. It was not consistent with our result due to the vast diversity of tissue property, however, it could be conclude collectively that traditional TCA/acetone methods was not very effective at protein extraction of complex tissues including LD, in spite of TCA/acetone or TCA/acetone-B.

Taken together, our modified TCA/acetone method worked very well in LD protein extraction, yielding the highest quality of 2-DE gel as well as displaying lower variation between replicates. The result could be explained as follows: additional grinding the hard centrifugation precipitates produced fine powder, enlarging contact area between precipitates and washing solution. Meanwhile, 90% acetone enhanced capacity of the cleanup solvent to dissolve the non-protein substance. Therefore, the above two steps promoted the removal of contaminants. Additionally, it stood to reason that the fine powder obtained through air-drying could expose more sample surface to lysis buffer, facilitating protein re-dissolution. Noteworthy, detected proteins of TCA/acetone-G-W sharing very small part of the total estimated proteome maybe were not more than sequential extraction based on lysis buffer whose procedures were too complex to perform comparative proteomics analysis containing a large number of samples [22,49]. In the current study, what was mainly focused on was the effectiveness of modified manipulation on sample preparation rather than other factors affecting 2-DE result. Continuous progress in buffer composition [50] and staining procedures [51] provide opportunities to achieve higher gel resolution. The protocol described here, combining with other optimized elements [52], might contribute to more accurate 2-DE analysis.

**Fig 2:**
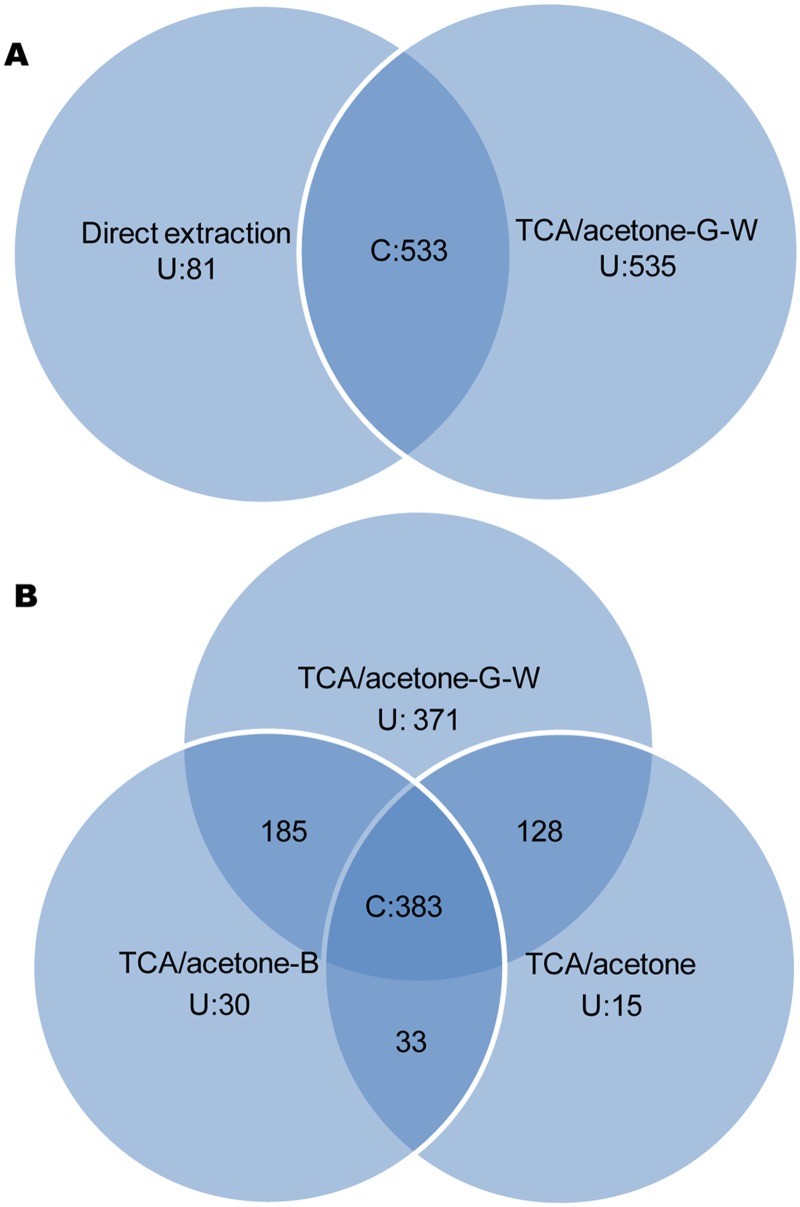
Overlay analysis of total protein spots with different methods. A: comparison of TCA/acetone-G-W to direct extraction; B: comparison of TCA/acetone-G-W to two standard TCA/acetone procedures. U represented the number of unique spots from each method; C represented the number of spots common to all the methods.

To further understand our modified protocol, overlay analysis of TCA/acetone-G-W to direct extraction and to two standard TCA/acetone methods regarding spot pattern were conducted respectively. As shown in Fig 2A, there were 533 protein spots present in both direct extraction and TCA/acetone-G-W. The number of unique spots in direct extraction was 81, far less than TCA/acetone-G-W(535). The result wasn’t surprising, since many spots were covered owning to low resolution, high background, serious streaking and smearing in gels. When we analyzed spot pattern of direct extraction with PDQuest software, it was difficult to distinguish a real spot from streaking and smearing due to blurry edges, going against the following steps such as spot incision and identification with MS. Thus contaminant elimination was much necessary for 2-DE gel with high resolution and clean background in LD proteomics. Fig 2B delivered the comparison of protein spots between TCA/acetone methods. 383 protein spots existed in all the three procedures; 511 spots were common to spot patterns of TCA/acetone and TCA/acetone-G-W, which accounted for 91.4% of all spots with TCA/acetone. Parallelly, 568 spots simultaneously occurred in TCA/acetone-B and TCA/acetone-G-W, taking up 90.0% of all spots with TCA/acetone-B. The total number of unique proteins detected by TCA/acetone-G-W was the highest (371), far exceeding the corresponding number for TCA/acetone-B (30) and TCA/acetone (15). The above results implied that TCA/acetone-G-W not only captured the most of proteins present in common TCA/acetone extracts, but also extracted many novel proteins. The increasing number of detected protein spots should be attributed to better spot focusing and more extracted proteins in TCA/acetone-G-W.

In the following analysis, TCA/acetone and TCA/acetone-B but not direct extraction method served as controls, since 2-DE gel with direct extraction wasn’t suited for downstream analysis in proteomics, and may even lead to inaccurate result due to the interference of a great deal of streaking and smearing. In S1 Fig, total proteins with three TCA/acetone methods were categorized according to molecular weight and normalized volume. TCA/acetone-G-W produced the most protein spots in all the categories of molecular weight, and was also favored by total protein spots with normalized volume < 1000. Differential proteins were classified based on normalized volume (S2 Fig), out of which highest intensitive spots in TCA/acetone-G-W were much more than standard TCA/acetone procedures in the normalized volume ranges < 1000. In general, proteins with high molecular weight are prone to precipitate with difficulty to re-dissolve [21] and be absorbed by strips. Meanwhile, low abundance proteins are susceptible to loss but not visualization [2,53]. While in our study, TCA/acetone-G-W approach provided a clearer 2-DE gel with more high molecular weight proteins and more low abundance proteins. A possible explanation could be that the modified approach facilitated the extraction or detection of these proteins. Previous studies have shown that the TCA/acetone precipitation can promote enrichment of basic proteins [23,31]. Indeed there were more protein spots in basic region than acidic region based on our result (Fig 1), but it was also possible that that initial sample contained more basic proteins than acidic.

In previous studies, physiochemical heterogeneity of proteins has been deemed to be the basis of substantially different protein extracts between methods. Accordingly, MS analysis has been performed to investigate hydropathicity, post-translational modifications, subcellular localization and proteolysis of differential proteins [19,21,24]. However, it failed to reveal any clear patterns that might account for protein difference between methods, implying the complexity of protein extraction. In our study, the diversity of physical means resulted in change of protein extracts, indicating the importance of physical means in protein extraction. Therefore, it was necessary to concentrate on physical means as well as protein property when explaining protein difference between extraction methods. In the past, physical techniques have been concerned and adopted in protein extraction including sonication, freeze-thaw and high speed blending [45]. In addition, Islam et al. [23] have performed a similar methodology by grinding precipitates after air-drying. To our knowledge, grinding precipitate between washing and dissolution assisted in protein solubilization, but not in contaminant removal. When we treated LD samples as described by Islam et al., the voltage failed to increase to the preset volts (10,000V) during IEF, indicating interference of residual contaminant.

Discriminant analysis, an important multivariate statistical method, usually used to verify whether a number of observations belong to different groups [19,54], has increasingly been employed in 2-DE analysis. In the present study, a Multiple Discriminant Analysis (MDA) was carried out to test if there was significant difference in protein profile between protocols. S1 Table showed the parameters of six protein spots, which had generated two canonical discriminant functions, making a successful classification. First 2 canonical discriminant functions were used in the analysis, with the first one explaining 79.6% of the total variance and the second accounting for 20.4% (S2 Table). S3 Fig displayed the predicted categories of various observations and separation of the three groups based on the canonical discriminant functions. The predicted group membership accorded with the original, indicating a 100% correct grouping of the original gels into their corresponding extraction methods. Nevertheless the cross-validation only showed 88.9% of accuracy in the re-matching stage (Table 2). One actual case of TCA/acetone was classified as TCA/acetone-B by mistake. We were not sure if this was an experiment error or a signal for less gel difference between TCA/acetone and TCA/acetone-B, or else, it could also be explained with the poor repeatability of TCA/acetone.

**Table 2:** Classification and cross-validation results.

		Method	Predicted Group Membership			Total
			TCA/acetone-G-W	TCA/acetone-B	TCA/acetone	
Original	Count	TCA/acetone-G-W	3	0	0	3
		TCA/acetone-B	0	3	0	3
		TCA/acetone	0	0	3	3
	%	TCA/acetone-G-W	100.0	.0	.0	100.0
		TCA/acetone-B	.0	100.0	.0	100.0
		TCA/acetone	.0	.0	100.0	100.0
Cross-validated	Count	TCA/acetone-G-W	3	0	0	3
		TCA/acetone-B	0	3	0	3
		TCA/acetone	0	1	2	3
	%	TCA/acetone-G-W	100.0	.0	.0	100.0
		TCA/acetone-B	.0	100.0	.0	100.0
		TCA/acetone	.0	33.3	66.7	100.0

100.0% of original grouped cases were correctly classified, and 88.9% of cross-validated grouped cases were correctly classified.

In TCA/acetone method, fully drying of precipitates can improve protein quality, but over drying may affect protein re-dissolution. Previous studies have demonstrated that TCA/acetone precipitates should be air-dried for no more than 10 min to avoid over drying. However, LD protein precipitates were still wet after 10 min air-drying (20°C). In order to fully dry LD precipitates, the drying period was extend to 30 min, and the comparison to 10 min was also performed on protein yield and 2-DE profile. Precipitated protein mass and protein concentration were shown in Table 1. It was found that 10 min air-drying produced significantly greater protein precipitates than 30 min (Table 1, TCA/acetone-G-W). However, it did not mean that the 10 min air-drying gained more protein in view of residual acetone or water in pellets. There was no significant difference between protein concentrations (TCA/acetone-G-W and 10min air-drying in Table 1), suggesting that prolonged air-drying didn’t lead to severe protein loss. As to the 2-DE analysis, comparable results were delivered with no profound deviations, while in the partial regions of 2-DE gel, extended air-drying (30 min) showed an elevated background and minimal horizontal streaking and smearing (S4 Fig in red dashed box) due to full drying. Therefore, we considered that the length of air-drying precipitate mainly depended on sample property and the actual parameters in laboratory which needed to be adapted to tissue sample before the final experiment.

## Conclusions

TCA/acetone precipitation is absolutely necessary for protein extraction of LD with high levels of lipids and nucleic acids. However, the solid precipitate is hard to be dispersed, restricting contaminant removal and protein re-dissolution. Accordingly, we modified the traditional TCA/acetone method as follows: i) grinded protein precipitates in pre-chilled acetone to emulsion with a glass tissue grinder; ii) adopted 90% acetone as washing solution. Our results reported that modifications facilitated removal of non-protein contaminants and protein solubilization, ultimately improving 2-DE pattern. The additional steps may seem labor-consuming, however, the improved 2-DE gel quality far outweighed the small additional labor investment in protein extraction. Furthermore, by comparing 30 min with 10 min air-drying, we observed that the former did not reduce the protein solubility; conversely it made 2-DE gel with less streaking and smearing. To sum up, our findings improved efficiency of TCA/acetone precipitation, providing a novel opportunity for comparative proteomic experiment of recalcitrant tissues.

## Supporting Information

S1 Fig Comparison of total protein distributions with different methods. A: Protein distribution according to molecular weight; B: Protein distribution according to normalized volume. Bars indicated the SD, and columns with different letters within each range corresponded to statistically significant differences (ANOVA, p < 0.05).
(TIF)

S2 Fig Classification of differential spots within different categories of normalized volume. Columns with different colors indicated the number of the differential spots, which had the highest intensity in the corresponding protocols.
(TIF)

S3 Fig Separation of extraction methods according to the canonical functions generated by the MDA. The cases were correctly classified and successfully separated by the two canonical discriminant functions.
(TIF)

S4 Fig 2-DE comparison between 30 min (A) and 10 min (B) air-drying LD proteins. The 30 min air-drying produced a better 2-DE gel, especially in red dashed boxes.
(TIF)

S1 Table Canonical discriminant function coefficients and normalized volumes of the six spots sufficient to differentiate between methods. (DOCX)

S2 Table Percentage of variance explained by the canonical functions generated in the MDA. (DOCX)
