# Supplementary figures and images for: An Optimized Trichloroacetic Acid/Acetone Precipitation Method for Two-Dimensional Gel Electrophoresis Analysis of Qinchuan Cattle Longissimus Dorsi Muscle Containing High Proportion of Marbling
Source: PLoS One. 2015 Apr 20;10(4):e0124723. doi: 10.1371/journal.pone.0124723 (PMC4404140; doi:10.1371/journal.pone.0124723)

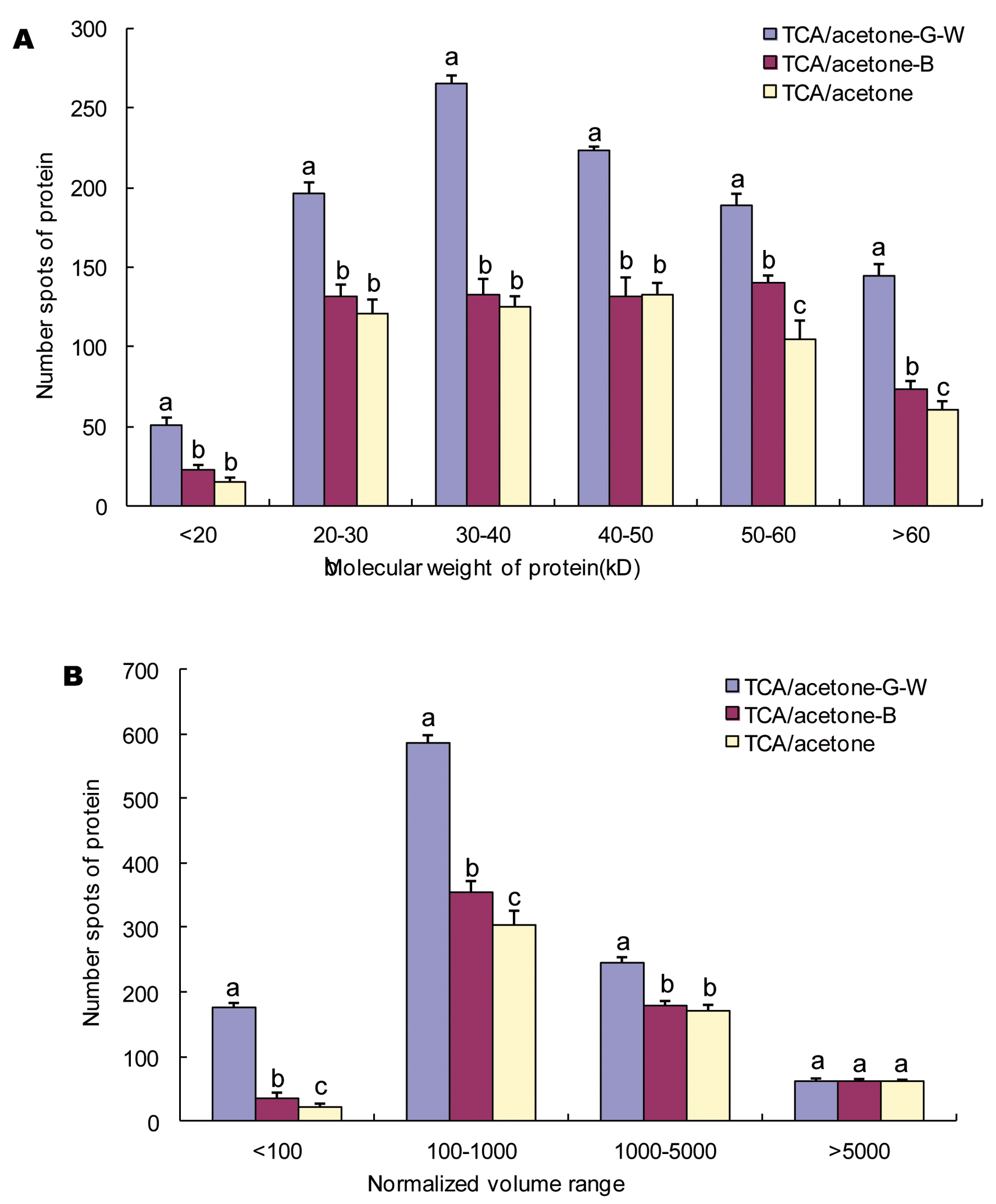

Supplement: S1 Fig — A: Protein distribution according to molecular weight; B: Protein distribution according to normalized volume. Bars indicated the SD, and columns with different letters within each range corresponded to statistically significant differences (ANOVA, p < 0.05). (TIF) [file pone.0124723.s001.tif]

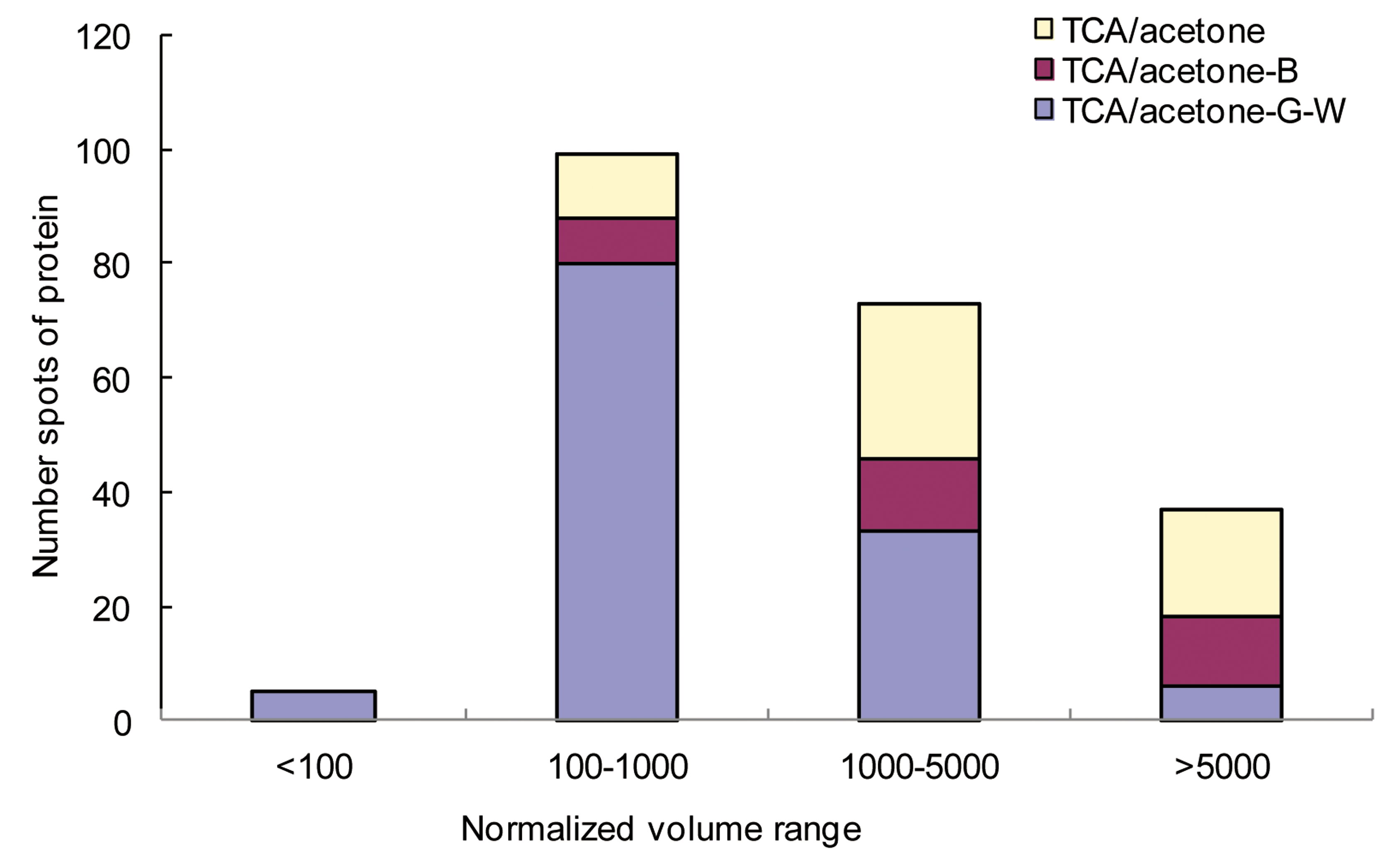

Supplement: S2 Fig — Columns with different colors indicated the number of the differential spots, which had the highest intensity in the corresponding protocols. (TIF) [file pone.0124723.s002.tif]

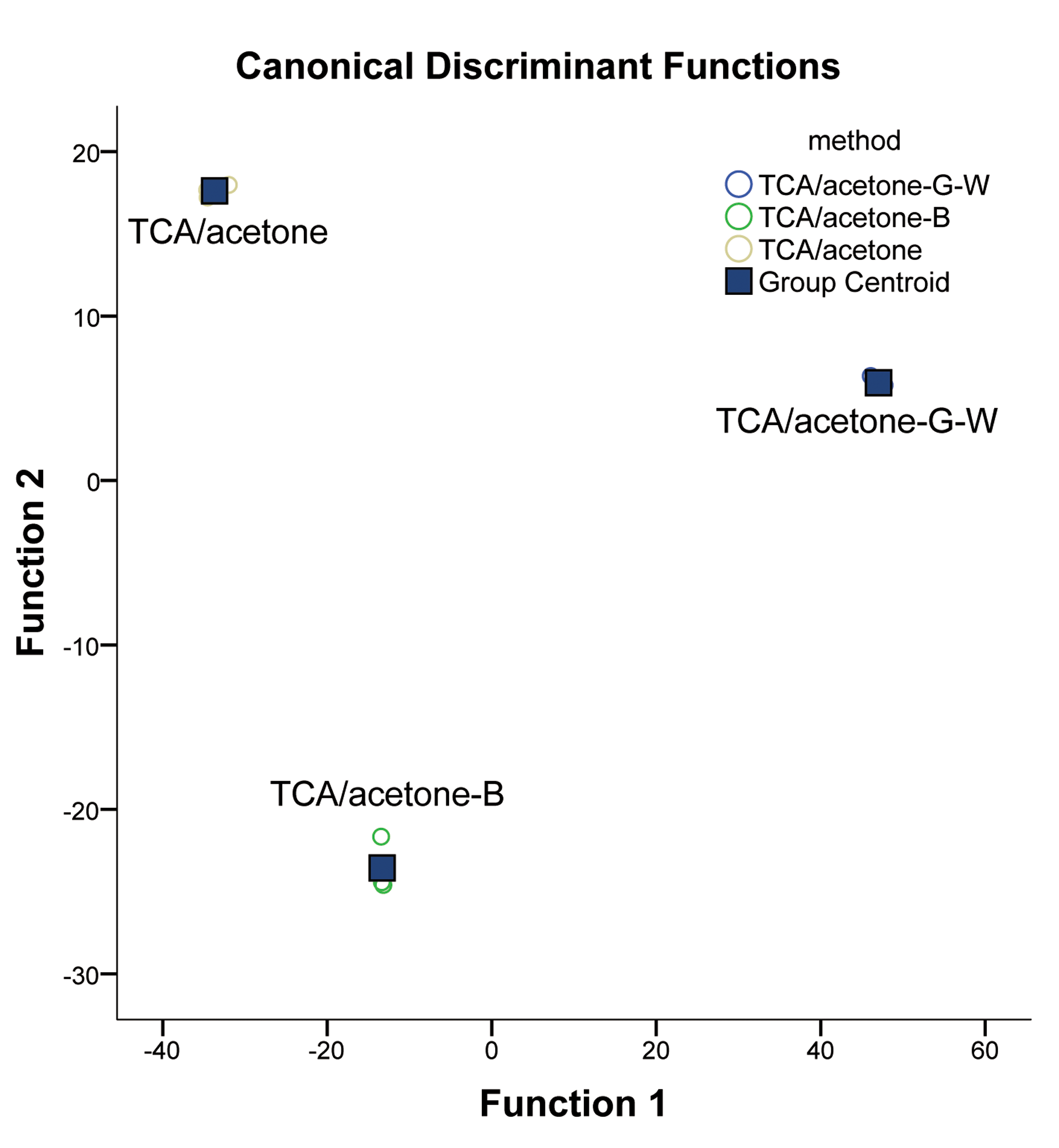

Supplement: S3 Fig — The cases were correctly classified and successfully separated by the two canonical discriminant functions. (TIF) [file pone.0124723.s003.tif]

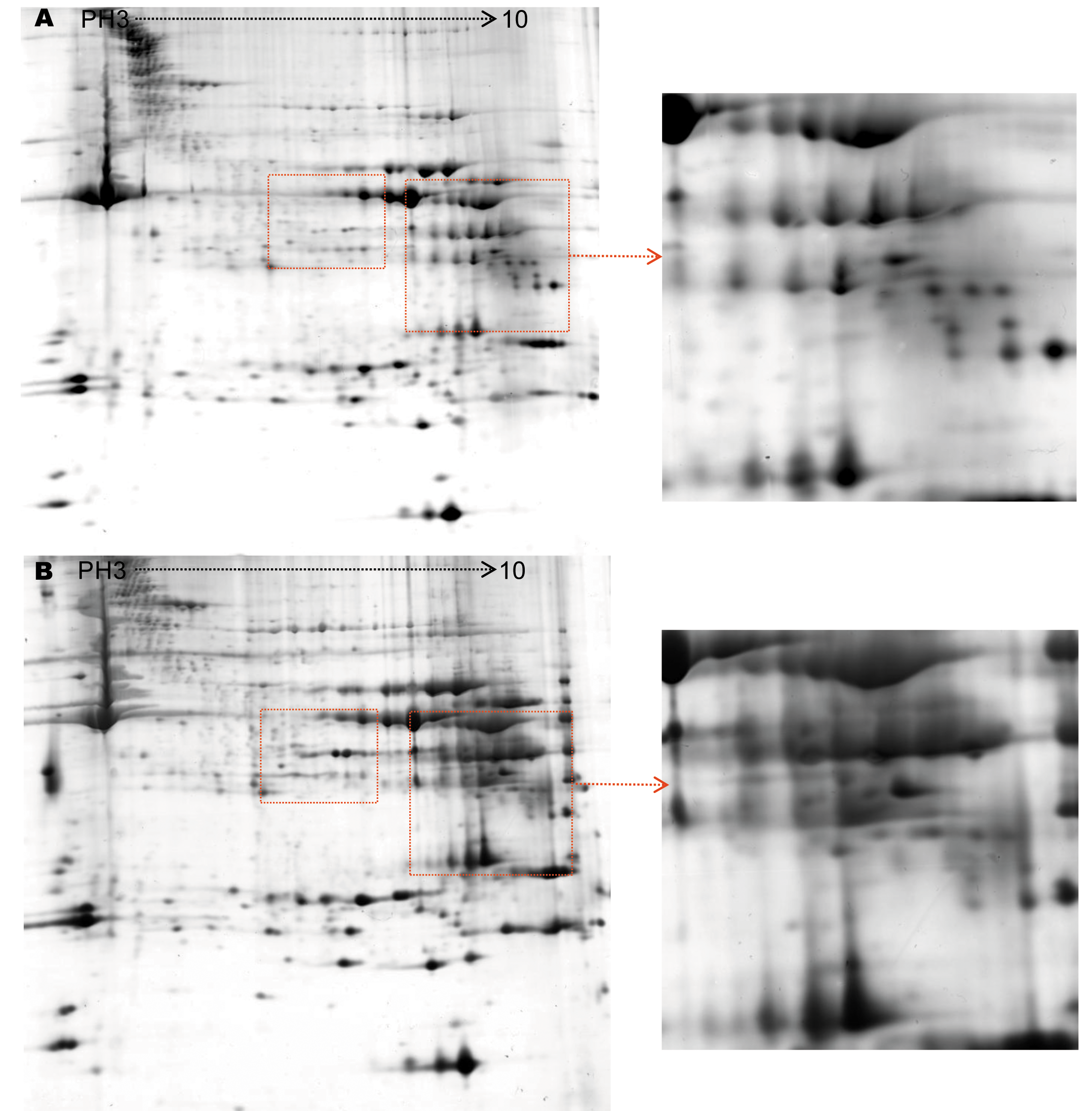

Supplement: S4 Fig — The 30 min air-drying produced a better 2-DE gel, especially in red dashed boxes. (TIF) [file pone.0124723.s004.tif]
