# Supplementary material for: An Optimized Trichloroacetic Acid/Acetone Precipitation Method for Two-Dimensional Gel Electrophoresis Analysis of Qinchuan Cattle Longissimus Dorsi Muscle Containing High Proportion of Marbling
Source: PLoS One. 2015 Apr 20;10(4):e0124723. doi: 10.1371/journal.pone.0124723 (PMC4404140; doi:10.1371/journal.pone.0124723)
Supplement: S1 Table — (DOCX) [file pone.0124723.s005.docx]

S1 Table. Canonical discriminant function coefficients and normalized volumes of the six spots sufficient to differentiate between methods.

| spot | Function | | Normalized volume | | | | | |
| --- | --- | --- | --- | --- | --- | --- | --- | --- |
|  | 1 | 2 | TCA/acetone-G-W | | TCA/acetone-B | | TCA/acetone | |
|  |  |  | Mean ± SD | C.V. | Mean ± SD | C.V. | Mean ± SD | C.V. |
| S2509 | 4.113 | 2.994 | 2115.3 ±388.9 | 0.18 | 773.3 ±156.3 | 0.20 | 3303.3 ±721.8 | 0.22 |
| S2524 | 9.452 | 4.471 | 245.0 ±41.7 | 0.17 | 104.7 ±44.6 | 0.43 | 93.3 ±36.1 | 0.39 |
| S2734 | 2.024 | -1.595 | 1061.7 ±194.5 | 0.18 | 2028.3 ±556.4 | 0.27 | 3225.3 ±680.5 | 0.21 |
| S3006 | 3.255 | 3.161 | 2412.0 ±336.9 | 0.14 | 459.3 ±178.5 | 0.39 | 1821.0 ±455.0 | 0.30 |
| S3110 | -12.94 | -1.037 | 1980.0 ±422.3 | 0.21 | 2298.0 ±397.5 | 0.17 | 5356.0 ±896.9 | 0.17 |
| S3114 | -2.35 | -7.472 | 442.3 ±24.4 | 0.06 | 346.0 ±53.1 | 0.15 | 204.7 ±54.2 | 0.26 |
|  |  |  |  | Min=0.06 |  | Min=0.15 |  | Min=0.17 |
|  |  |  |  | Max=0.21 |  | Max=0.43 |  | Max=0.39 |
|  |  |  |  | Average=0.16 |  | Average=0.27 |  | Average=0.26 |
|  |  |  |  | SD=0.05 |  | SD=0.12 |  | SD=0.08 |

Values of Canonical Discriminant Function Coefficients, normalized volume (mean ± SD) and coefficient of variance are presented.
