# Supplementary material for: An Optimized Trichloroacetic Acid/Acetone Precipitation Method for Two-Dimensional Gel Electrophoresis Analysis of Qinchuan Cattle Longissimus Dorsi Muscle Containing High Proportion of Marbling
Source: PLoS One. 2015 Apr 20;10(4):e0124723. doi: 10.1371/journal.pone.0124723 (PMC4404140; doi:10.1371/journal.pone.0124723)
Supplement: S2 Table — (DOCX) [file pone.0124723.s006.docx]

| Function | % of Variance | Cumulative % | Canonical Correlation |
| --- | --- | --- | --- |
| 1 | 79.6 | 79.6 | 1.000 |
| 2 | 20.4 | 100.0 | .999 |

S2 Table. Percentage of variance explained by the canonical functions generated in the MDA

First 2 canonical discriminant functions were used in the analysis, and explained 100% variance.
